# Supplementary material for: Transcriptome Analysis Reveals Vimentin-Induced Disruption of Cell–Cell Associations Augments Breast Cancer Cell Migration
Source: Cells. 2022 Dec 13;11(24):4035. doi: 10.3390/cells11244035 (PMC9776984; doi:10.3390/cells11244035)
Supplement: Supplementary file 1 [file cells-11-04035-s001.zip › DEGs and enrichment analysis.zip!/DEGs and enrichment analysis/WT_VIMvsVector KEGG/GSEA/WT_VIM/TASTE_TRANSDUCTION/TASTE_TRANSDUCTION(HSA04742).html]

Details for gene set TASTE\_TRANSDUCTION(HSA04742)[GSEA]

|  || Dataset | fpkm.sample |
| Phenotype | sample.cls |
| Upregulated in class | WT\_VIM |
| GeneSet | TASTE\_TRANSDUCTION(HSA04742) |
| Enrichment Score (ES) | 0.700061 |
| Normalized Enrichment Score (NES) | 1.1890854 |
| Nominal p-value | 0.0 |
| FDR q-value | 0.2491254 |
| FWER p-Value | 0.498 |
Table: GSEA Results Summary

  

Fig 1: Enrichment plot: TASTE\_TRANSDUCTION(HSA04742)      
 Profile of the Running ES Score & Positions of GeneSet Members on the Rank Ordered List

  

| PROBE | DESCRIPTION (from dataset) | GENE SYMBOL | GENE\_TITLE | RANK IN GENE LIST | RANK METRIC SCORE | RUNNING ES | CORE ENRICHMENT || 1 | ENSG00000255275 | AL080251.1 |  |  | 14 | Infinity | -0.0001 | Yes |
| 2 | ENSG00000011677 | GABRA3 |  |  | 48 | Infinity | -0.0005 | Yes |
| 3 | ENSG00000173662 | TAS1R1 |  |  | 108 | Infinity | -0.0014 | Yes |
| 4 | ENSG00000127362 | TAS2R3 |  |  | 122 | Infinity | -0.0015 | Yes |
| 5 | ENSG00000070985 | TRPM5 |  |  | 668 | Infinity | -0.0106 | Yes |
| 6 | ENSG00000274659 | LINC02371 |  |  | 975 | Infinity | -0.0156 | Yes |
| 7 | ENSG00000186912 | P2RY4 |  |  | 1026 | Infinity | -0.0164 | Yes |
| 8 | ENSG00000178394 | HTR1A |  |  | 1676 | Infinity | -0.0272 | Yes |
| 9 | ENSG00000137841 | PLCB2 |  |  | 2462 | 14.413 | 0.1485 | Yes |
| 10 | ENSG00000127588 | GNG13 |  |  | 2552 | 9.081 | 0.2661 | Yes |
| 11 | ENSG00000164626 | KCNK5 |  |  | 2902 | 5.316 | 0.3298 | Yes |
| 12 | ENSG00000123360 | PDE1B |  |  | 3349 | 3.899 | 0.3734 | Yes |
| 13 | ENSG00000111664 | GNB3 |  |  | 3421 | 3.719 | 0.4210 | Yes |
| 14 | ENSG00000185933 | CALHM1 |  |  | 3495 | 3.669 | 0.4679 | Yes |
| 15 | ENSG00000203523 | TAS2R2P |  |  | 3681 | 3.389 | 0.5092 | Yes |
| 16 | ENSG00000096433 | ITPR3 |  |  | 3818 | 3.258 | 0.5496 | Yes |
| 17 | ENSG00000168447 | SCNN1B |  |  | 4426 | 2.707 | 0.5748 | Yes |
| 18 | ENSG00000054179 | ENTPD2 |  |  | 4563 | 2.635 | 0.6071 | Yes |
| 19 | ENSG00000124493 | GRM4 |  |  | 5434 | 2.220 | 0.6214 | Yes |
| 20 | ENSG00000111319 | SCNN1A |  |  | 6423 | 1.940 | 0.6301 | Yes |
| 21 | ENSG00000187848 | P2RX2 |  |  | 6519 | 1.920 | 0.6537 | Yes |
| 22 | ENSG00000169962 | TAS1R3 |  |  | 6707 | 1.875 | 0.6751 | Yes |
| 23 | ENSG00000138622 | HCN4 |  |  | 8655 | 1.527 | 0.6622 | Yes |
| 24 | ENSG00000179546 | HTR1D |  |  | 9619 | 1.402 | 0.6642 | Yes |
| 25 | ENSG00000214415 | GNAT3 |  |  | 10463 | 1.318 | 0.6672 | Yes |
| 26 | ENSG00000174233 | ADCY6 |  |  | 11257 | 1.241 | 0.6701 | Yes |
| 27 | ENSG00000072062 | PRKACA |  |  | 11318 | 1.236 | 0.6853 | Yes |
| 28 | ENSG00000129467 | ADCY4 |  |  | 11398 | 1.230 | 0.7001 | Yes |
| 29 | ENSG00000166828 | SCNN1G |  |  | 16865 | 0.865 | 0.6188 | No |
| 30 | ENSG00000141837 | CACNA1A |  |  | 17148 | 0.848 | 0.6252 | No |
| 31 | ENSG00000127366 | TAS2R5 |  |  | 18996 | 0.743 | 0.6036 | No |
| 32 | ENSG00000169860 | P2RY1 |  |  | 19518 | 0.714 | 0.6041 | No |
| 33 | ENSG00000277481 | PKD1L3 |  |  | 20578 | 0.657 | 0.5948 | No |
| 34 | ENSG00000236283 | AC019197.1 |  |  | 20958 | 0.637 | 0.5968 | No |
| 35 | ENSG00000142875 | PRKACB |  |  | 21908 | 0.582 | 0.5883 | No |
| 36 | ENSG00000168830 | HTR1E |  |  | 24196 | 0.421 | 0.5551 | No |
| 37 | ENSG00000270923 | TAS2R6P |  |  | 24356 | 0.410 | 0.5578 | No |
| 38 | ENSG00000115252 | PDE1A |  |  | 24849 | 0.367 | 0.5543 | No |
| 39 | ENSG00000136531 | SCN2A |  |  | 25466 | 0.310 | 0.5479 | No |
| 40 | ENSG00000152822 | GRM1 |  |  | 25513 | 0.307 | 0.5511 | No |
| 41 | ENSG00000127364 | TAS2R4 |  |  | 25771 | 0.274 | 0.5504 | No |
| 42 | ENSG00000151067 | CACNA1C |  |  | 26541 | 0.186 | 0.5398 | No |
| 43 | ENSG00000136928 | GABBR2 |  |  | 26560 | 0.185 | 0.5419 | No |
| 44 | ENSG00000179097 | HTR1F |  |  | 27110 | 0.086 | 0.5337 | No |
| 45 | ENSG00000135312 | HTR1B |  |  | 29795 | 0.000 | 0.4883 | No |
| 46 | novel.613 | - |  |  | 30166 | 0.000 | 0.4820 | No |
| 47 | ENSG00000153253 | SCN3A |  |  | 30177 | 0.000 | 0.4818 | No |
| 48 | ENSG00000186038 | HTR3E |  |  | 30407 | 0.000 | 0.4779 | No |
| 49 | ENSG00000230483 | AC124057.1 |  |  | 31685 | NaN | 0.4564 | No |
| 50 | ENSG00000169432 | SCN9A |  |  | 31762 | NaN | 0.4553 | No |
| 51 | ENSG00000151834 | GABRA2 |  |  | 31866 | NaN | 0.4537 | No |
| 52 | ENSG00000178084 | HTR3C |  |  | 32298 | NaN | 0.4465 | No |
| 53 | ENSG00000256436 | TAS2R31 |  |  | 32768 | NaN | 0.4387 | No |
| 54 | ENSG00000121381 | TAS2R9 |  |  | 33020 | NaN | 0.4346 | No |
| 55 | ENSG00000107593 | PKD2L1 |  |  | 33279 | NaN | 0.4303 | No |
| 56 | ENSG00000256274 | TAS2R64P |  |  | 33659 | NaN | 0.4240 | No |
| 57 | ENSG00000256271 | CACNA1C-AS2 |  |  | 33660 | NaN | 0.4242 | No |
| 58 | ENSG00000109158 | GABRA4 |  |  | 33826 | NaN | 0.4215 | No |
| 59 | ENSG00000108684 | ASIC2 |  |  | 33854 | NaN | 0.4212 | No |
| 60 | ENSG00000022355 | GABRA1 |  |  | 36127 | NaN | 0.3828 | No |
| 61 | ENSG00000256981 | AC134349.2 |  |  | 37115 | NaN | 0.3662 | No |
| 62 | ENSG00000185899 | TAS2R60 |  |  | 38041 | NaN | 0.3507 | No |
| 63 | ENSG00000253992 | AC103726.2 |  |  | 38148 | NaN | 0.3490 | No |
| 64 | ENSG00000186297 | GABRA5 |  |  | 38338 | NaN | 0.3460 | No |
| 65 | ENSG00000236398 | TAS2R39 |  |  | 38393 | NaN | 0.3452 | No |
| 66 | ENSG00000166736 | HTR3A |  |  | 38481 | NaN | 0.3438 | No |
| 67 | ENSG00000204681 | GABBR1 |  |  | 38781 | NaN | 0.3389 | No |
| 68 | ENSG00000234066 | TAS2R62P |  |  | 40127 | NaN | 0.3162 | No |
| 69 | ENSG00000253403 | AC091944.1 |  |  | 41104 | NaN | 0.2998 | No |
| 70 | ENSG00000256682 | AC006518.2 |  |  | 41204 | NaN | 0.2983 | No |
| 71 | ENSG00000256657 | AC244131.1 |  |  | 41309 | NaN | 0.2967 | No |
| 72 | ENSG00000212125 | TAS2R15P |  |  | 41985 | NaN | 0.2854 | No |
| 73 | ENSG00000263571 | AC004147.2 |  |  | 42786 | NaN | 0.2719 | No |
| 74 | ENSG00000257138 | TAS2R38 |  |  | 43369 | NaN | 0.2622 | No |
| 75 | ENSG00000186136 | TAS2R42 |  |  | 43586 | NaN | 0.2587 | No |
| 76 | ENSG00000154678 | PDE1C |  |  | 43835 | NaN | 0.2546 | No |
| 77 | ENSG00000121318 | TAS2R10 |  |  | 43880 | NaN | 0.2540 | No |
| 78 | ENSG00000256651 | AC006518.1 |  |  | 44481 | NaN | 0.2440 | No |
| 79 | ENSG00000268170 | AC073342.2 |  |  | 45199 | NaN | 0.2319 | No |
| 80 | ENSG00000121377 | TAS2R7 |  |  | 45455 | NaN | 0.2278 | No |
| 81 | ENSG00000128519 | TAS2R16 |  |  | 45830 | NaN | 0.2215 | No |
| 82 | ENSG00000221937 | TAS2R40 |  |  | 46707 | NaN | 0.2068 | No |
| 83 | ENSG00000256019 | TAS2R63P |  |  | 47147 | NaN | 0.1995 | No |
| 84 | ENSG00000275811 | HTR1DP1 |  |  | 47843 | NaN | 0.1879 | No |
| 85 | ENSG00000179002 | TAS1R2 |  |  | 48013 | NaN | 0.1852 | No |
| 86 | ENSG00000255837 | TAS2R20 |  |  | 48048 | NaN | 0.1847 | No |
| 87 | ENSG00000145863 | GABRA6 |  |  | 48499 | NaN | 0.1772 | No |
| 88 | ENSG00000212128 | TAS2R13 |  |  | 48995 | NaN | 0.1690 | No |
| 89 | ENSG00000212124 | TAS2R19 |  |  | 48996 | NaN | 0.1691 | No |
| 90 | ENSG00000212126 | TAS2R50 |  |  | 48997 | NaN | 0.1692 | No |
| 91 | ENSG00000186090 | HTR3D |  |  | 49326 | NaN | 0.1638 | No |
| 92 | ENSG00000236107 | AC010127.1 |  |  | 49523 | NaN | 0.1606 | No |
| 93 | ENSG00000121314 | TAS2R8 |  |  | 50884 | NaN | 0.1377 | No |
| 94 | ENSG00000238020 | HTR3E-AS1 |  |  | 50992 | NaN | 0.1360 | No |
| 95 | ENSG00000221855 | TAS2R41 |  |  | 51750 | NaN | 0.1233 | No |
| 96 | ENSG00000155897 | ADCY8 |  |  | 52963 | NaN | 0.1029 | No |
| 97 | ENSG00000109991 | P2RX3 |  |  | 54589 | NaN | 0.0755 | No |
| 98 | ENSG00000277705 | AC007450.3 |  |  | 54854 | NaN | 0.0712 | No |
| 99 | ENSG00000266535 | AC008133.1 |  |  | 55279 | NaN | 0.0641 | No |
| 100 | ENSG00000165059 | PRKACG |  |  | 55991 | NaN | 0.0522 | No |
| 101 | ENSG00000169777 | TAS2R1 |  |  | 56067 | NaN | 0.0511 | No |
| 102 | ENSG00000149305 | HTR3B |  |  | 56635 | NaN | 0.0416 | No |
| 103 | ENSG00000246627 | CACNA1C-AS1 |  |  | 58163 | NaN | 0.0159 | No |
| 104 | ENSG00000226761 | TAS2R46 |  |  | 58181 | NaN | 0.0157 | No |
| 105 | ENSG00000256188 | TAS2R30 |  |  | 58525 | NaN | 0.0100 | No |
| 106 | ENSG00000255374 | TAS2R43 |  |  | 58565 | NaN | 0.0095 | No |
| 107 | ENSG00000233576 | AC131235.2 |  |  | 59122 | NaN | 0.0002 | No |
Table: GSEA details [plain text format]

  

Fig 2: TASTE\_TRANSDUCTION(HSA04742)      
 Blue-Pink O' Gram in the Space of the Analyzed GeneSet

  

Fig 3: TASTE\_TRANSDUCTION(HSA04742): Random ES distribution      
 Gene set null distribution of ES for **TASTE\_TRANSDUCTION(HSA04742)**
